# Optimization of Solvent-Free Microwave-Assisted Hydrodiffusion and Gravity Extraction of *Morus nigra* L. Fruits Maximizing Polyphenols, Sugar Content, and Biological Activities Using Central Composite Design

**DOI:** 10.3390/ph15010099

**Published:** 2022-01-14

**Authors:** Ahmed M. Mustafa, Eugenia Mazzara, Doaa Abouelenein, Simone Angeloni, Sonia Nunez, Gianni Sagratini, Víctor López, Marco Cespi, Sauro Vittori, Giovanni Caprioli, Filippo Maggi

**Affiliations:** 1 School of Pharmacy, Chemistry Interdisciplinary Project (ChIP), University of Camerino, 62032 Camerino, Italy; ahmed.mustafa@unicam.it (A.M.M.); eugenia.mazzara@unicam.it (E.M.); doaa.abouelenein@unicam.it (D.A.); simone.angeloni@unicam.it (S.A.); gianni.sagratini@unicam.it (G.S.); marco.cespi@unicam.it (M.C.); sauro.vittori@unicam.it (S.V.); giovanni.caprioli@unicam.it (G.C.); 2 Department of Pharmacognosy, Faculty of Pharmacy, Zagazig University, Zagazig 44519, Egypt; 3 Department of Pharmacy, Faculty of Health Sciences, Universidad San Jorge, Villanueva de Gállego, 50830 Zaragoza, Spain; snunez@usj.es (S.N.); ilopez@usj.es (V.L.); 4 Instituto Agroalimentario de Aragón-IA2, CITA-Universidad de Zaragoza, 50830 Zaragoza, Spain

**Keywords:** *Morus nigra*, microwave hydrodiffusion and gravity, polyphenols, sugar content, *α*-glucosidase, lipase, xanthine oxidase, DPPH radical scavenging, central composite design

## Abstract

Black mulberry, *Morus nigra* L. (family: Moraceae), is a healthy food and medicinal plant. Microwave hydrodiffusion and gravity (MHG) is one of the most innovative applications of solvent-free microwave extraction. The aim of this study was to optimize for the first time the MHG solvent-free extraction of polyphenols and sugars from *M. nigra* fruits. Optimization was carried out using a central composite design (CCD) with selected responses such as extraction yield, total polyphenol (TPC), flavonoid (TFC), anthocyanin (TAC), and sugar (TSC) contents, in addition to DPPH radical scavenging, and α-glucosidase (AGHi), lipase (Li), and xanthine oxidase (XOi) inhibition as tools to evaluate the best parameters for efficient and rapid extraction of black mulberry. The optimized extract was characterized in terms of the aforementioned parameters to validate the models, and was further analyzed for 36 individual polyphenols using HPLC-MS/MS. The optimized MHG extract was finally compared with traditional extracts, and demonstrated much better performance in terms of TPC, TAC, and Li, while the traditional extracts showed better XOi and AGHi. In conclusion, MHG is a valuable green technique for the production of non-degraded black mulberry polyphenol-rich extract and we suggest its larger use in the pharmaceutical and food industries.

## 1. Introduction

The black mulberry (*Morus nigra* L.) is a Moraceae family plant that is used as a food and medicine. It belongs to the genus *Morus* and is one of its most important species. This species is well-known for its nutritional value and unusual flavor, as well as being a good source of numerous bioactive phytonutrients [1]. The fruit of the black mulberry tree contains a high concentration of beneficial bioactive metabolites, such as phenolic compounds, which may have beneficial health effects [2]. Phenolic acids, flavonols, and anthocyanins are the three major categories of phenolic compounds contained in black mulberry. Phenolic acids include chlorogenic, gallic, syringic, neochlorogenic, and caffeic acids. Flavonols encompass quercetin-3-glucoside, rutin, and quercetin-3-malonylglucoside [3,4]. Anthocyanins comprise cyanidin-3-*O*-glucoside, cyanidin-3-*O*-rutinoside (the major ones), cyanidin-3-*O*-(6-malonyl-glucoside), and cyanidin-3-*O*-(6-dioxalyl-glucoside) (minor components) [5]. The mulberry fruit, in addition to being a food item, has been used in folk medicine for thousands of years, particularly in China, to cure sore throats, anemia, and tonsillitis [6]. It has been found to have a wide range of bioactivities, including free-radical scavenging [7,8], antidiabetic [9], neuroprotective [10], antifatigue [11], antiatherosclerosis [12], and immune-modulating [13], thanks to its high level of bioactive polyphenols.

The rising demand for mulberries, along with the fact that polyphenols and sugars found in these fruits are beneficial to human health, necessitates the development of quick and reliable methods for extracting these chemicals. Novel approaches such as supercritical fluid extraction (SFE) or ultrasound-assisted extraction (UAE) are effective alternatives to traditional methods, which possess drawbacks and frequently violate eco-extraction principles [14]. The only disadvantage of UAE is the use of an organic solvent, whereas SFE devices require a significant financial investment that most laboratories cannot afford. Microwave-assisted extraction (MAE) is another novel technology that has been shown to be particularly effective for essential oils hydrodistillation [15] and is being studied at several levels for polyphenols extraction [16,17]. Microwave volumetric heating with superior temperature control eliminates gradients and, as a result, avoids thermal deterioration. MAE, like the other cutting-edge techniques mentioned above, seeks more environmentally friendly protocols [16].

Microwave hydrodiffusion and gravity (MHG) is one of the most revolutionary applications of solvent-free microwave extraction. This technique extends the MAE’s boundaries even further in the direction of a greener process [18]. It extracts hydrophilic phytoconstituents from the matrix in the reactor using “in situ” water. It also weakens the matrix tissue and ruptures the phytochemical storage cavities. The in situ water in the fresh matrix is heated and swept outside of the plant tissues, carrying bioactive compounds with it. The MHG technique has been mainly employed in laboratory-scale trials and has proven to be really useful for fresh matrices [16]. When compared with traditional extraction methods, determining the biological activity of *M. nigra* fruits utilizing novel and green extraction techniques such as MHG is a big challenge. The aim of this study was to develop an advanced solvent-free extraction method for black mulberry fruits using MHG while analyzing different biological activities. Optimization of the solvent-free MHG extraction system was carried out with selected responses such as (1) extraction yield %, (2) total phenolic content (TPC), (3) total flavonoid content (TFC), (4) total anthocyanin content (TAC), (5) total sugar content (TSC), (6) α-glucosidase inhibition (AGHi), (7) lipase inhibition (Li), (8) xanthine oxidase inhibition (XOi), and (9) DPPH radical scavenging as tools to evaluate the best parameters for efficient and rapid extraction of black mulberry. The optimized MHG extract was analyzed for 36 individual polyphenols using HPLC-MS/MS. To the best of our knowledge, this is the first time that an optimization of the MHG solvent-free extraction of polyphenols and sugars from black mulberry has been carried out using a central composite design (CCD) for maximizing the previously mentioned biological activities.

## 2. Results and Discussion

### 2.1. Design of the Experiments (DoE) Analysis

**Figure 1:**
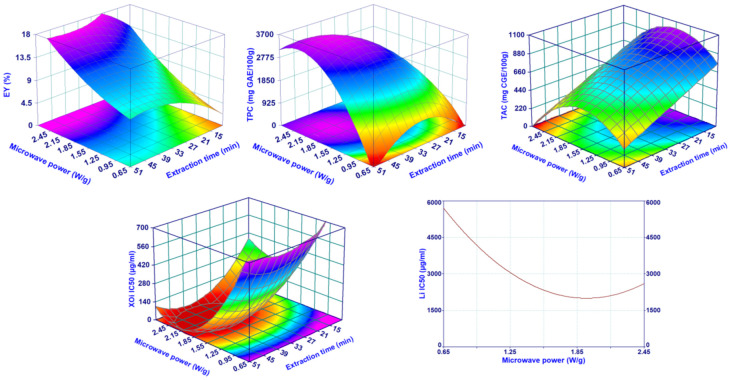
Surface plots showing the effect of the irradiation power and extraction time on the EY%, TPC, TAC, XOi, and Li.

The MHG analysis procedure, which employs a CCD approach, allows the creation of appropriate mathematical models able to describe how the extraction experimental conditions, namely, microwave power (MP) and extraction time (ET), influence the measured responses (EY%, TPC, TFC, TAC, TSC, AGHi, Li, XOi, and DPPH radical scavenging). All the results of the DoE analysis, the best models for each response, and the parameters used for their evaluation (R^2^_adj_, R^2^_pred,_ Mallows’ Cp statistic, *p*-values of the regression, and lack of fit) are reported in Table 1. Among all the responses considered, only the TSC and AGHi could not be described by the models, resulting in a non-significant regression. In addition, the modeling of TFC and DPPH, even if characterized by a statistically significant regression (in both cases the *p*-value was between 0.05 and 0.01), showed low values of R^2^_adj_ (around 0.5) and values of R^2^_pred_ lower than 0.27; thus, in this case, the DoE analysis can be used for a general understanding of the effect of MAE parameters but it cannot be used for predictions and optimization. All the other responses were well described by the full quadratic model or its reduced equations. In all cases, the regression was always statistically significant (highest *p*-values was for TPC equals to 0.005), with values of R^2^_adj_ and R^2^_pred_ always greater than 0.756 and 0.58, respectively. The defined models for all the responses that were efficiently described by the regression analysis (EY%, TPC, TAC, Li, XOi) are plotted in Figure 1 using the surface plots, with the exception of Li, which resulted dependent only on the MP, thus requiring a standard 2-D graph.

For EY%, TPC, and XOi, the DoE analysis indicated that best results (highest EY% and TPC, lowest XOi) were obtained operating at similar extraction conditions, namely, high microwave extraction power and long extraction time. The result was not surprising for the yield; in fact, it is consistent with those for microwave juice extraction from fresh fruits or vegetables [16,19] as well as for microwave extraction of essential oil [15,20]. The literature generally reported results aligned with those reported here for TPC. For example, Périno et al. reported an increase of TPC during MHG extraction of lettuce, operating at higher microwave power for a certain time [16], while Koyu et al. did not find an appreciable MP and ET effect on the juice obtained from *M. nigra* fruits [21]. In the last case, the results were difficult to compare with those obtained here, due to the highly different experimental setup used. In fact, the amount of extracted matrix was around 100 times less and the power, expressed as W/g, was consequently much higher, from 300 to 700 times. Regarding the XOi, there are no studies focusing on the effect of MAE parameters on XOi activity of aqueous extracts. DoE results suggest that high values of MP and long ET determine a higher extraction of polyphenols and compounds with inhibitory activity on XO rather than a selective extraction of these substances, as confirmed also by a certain correlation, statistically significant, between TPC and XOi with EY (%) (Pearson r equal to 0.616 and −0.762 and *p*-value of 0.044 and 0.006 for TPC and XOi, respectively). The lipase inhibition activity of the mulberry extract followed a similar trend as that of TPC and XOi but only for the MP, while the ET did not result to be a relevant parameter. Finally, TAC showed a completely different behavior. The amount of extracted anthocyanins increased by operating at medium-low MP, although the most important parameter was represented by the ET and, specifically as the extraction progressed, the TAC strongly reduced. This behavior suggests that anthocyanins are sensitive to high temperatures (high MP determines a faster rise of temperature in the extraction vessel) when those are maintained for long times. These results were in agreement with the literature [21,22,23,24], although a direct comparison of the TAC values was impossible due to the very different experimental setup, applied conditions, and operative parameters.

### 2.2. MHG Optimization and Model Validation

**Figure 2:**
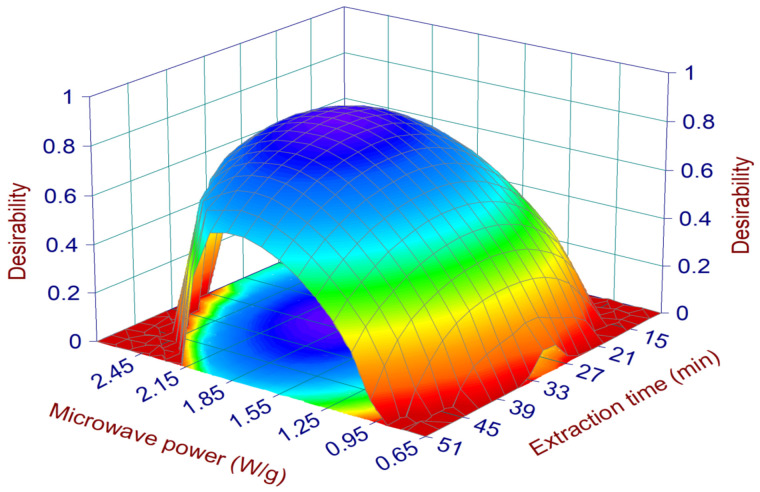
Surface plots of the desirability. The plots show the effect of microwave irradiation power and extraction time.

**Figure 3:**
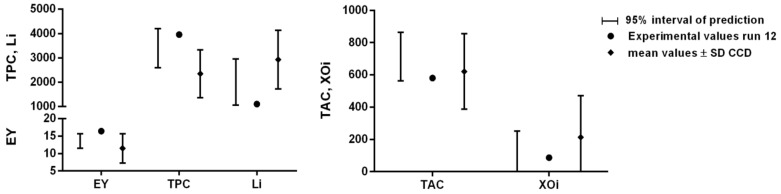
The features of the validation run (run 12) concerning the optimized responses. The predicted results are reported as predicted value and 95% interval of prediction.

Once the mathematical relationships between the operative parameters and each single response (EY%, TPC, TAC, Li and XOi) were defined, it was possible to optimize the MHG extraction process, i.e., define the extraction conditions capable of generating the best results for all responses at the same time. For this purpose, we chose the desirability approach, set in order to maximize EY%, TPC, and TAC, while the Li and XOi, which are represented by IC_50_ values, were minimized. The surface map of all the calculated D values is reported in Figure 2, showing the highest desirability values are located in the middle of the experimental domain, specifically in the ranges of 1.5–2.3 W/g and 16–44 min for the MP and ET, respectively (within these ranges the D values were always higher than 0.7). The conditions assuring the highest D (0.83) together with the 95% interval of confidence (95% CI) and prediction (95% PI) for each optimized response are reported in Table 2. These conditions were applied in run 12 (validation run) and the obtained juice was characterized in terms of the optimized responses (EY%, TPC, TAC, Li, and XOi) as well as in terms of the responses poorly described by the models (i.e., TFC, TSC, AGHi, and DPPH radical scavenging) and not used for the optimization step. The features of run 12 concerning the optimized responses are reported in Figure 3. The values of EY%, TPC, Li, TAC, and XOi were all within the range of the 95% interval of prediction, suggesting the reliability of the defined models. In addition, the absolute values were always higher than the average ones calculated for the CCD runs (Table 3), confirming that run 12 performed better than all the other runs if all these features were considered together. The only exception was represented by the TAC value, which was closer to the average value of the CCD runs. According to the model (surface plot of TAC in Figure 1), the TAC values suddenly fell when the MP reached values around 1.8–1.9 W/g (the MP value of the run 12). In this situation, small errors on the model prediction ability (which is always present, since it will never exactly describe the response variation within the experimental domain) could determine relevant changes of the response. The results of the responses not used for the optimization (Figure S1) are reported in the Supplementary Materials and, as expected, their absolute values were all close to the average values and within the standard deviation interval of the CCD runs.

### 2.3. Application of the HPLC-MS/MS Method to the Validated MHG Run

After optimizing the acquisition settings, target compound quantification was carried out, and the selected ion transitions and mass spectrometer parameters for each molecule are listed in Table S1. Similar to our previous work [25], linearity, limits of quantification (LOQs), limits of detection (LODs), and precision were utilized to validate the HPLC-MS/MS method before analysis of the validated MHG extract (Table S2). Figure S2 shows the HPLC-MS/MS chromatogram of a standard mixture of the 36 analytes shown as an overlapping MRM transition for each monitored compound.

Since polyphenols can be degraded by high temperature that is usually applied during MHG, the phenolic profiles of samples were analyzed by HPLC-MS/MS to confirm the retention of individual polyphenols. HPLC-MS/MS analysis was carried out for the quantitative determination of 36 bioactive phenolic components in the extract of the validated MHG run (i.e., run 12). A total of 23 phenolic compounds was detected, and their contents are given in Table 4. These results were in agreement with previous findings in black mulberry [4,26,27,28,29]. The results demonstrated that the extract of the validated MHG holds a potential for the tested biological activities because it contains a wide range of bioactive amounts of anthocyanins, phenolic acids, flavonols, flavan−3-ols, and dihydrochalcones.

### 2.4. Comparison between MHG Validation Run Extract and Conventional Solvent Extracts

**Figure 4:**
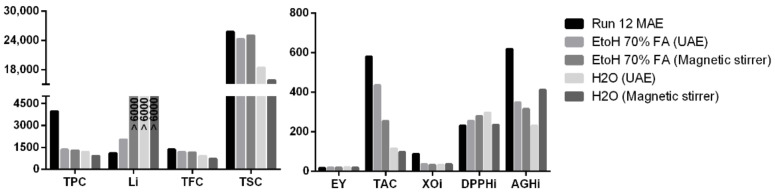
Comparison between selected responses in extracts obtained by MHG and conventional solvent extraction methods.

The results of MAE extraction appeared to be different from those obtained using conventional methods (ultrasound and magnetic stirrer). Specifically, the two methods produced comparable results only in terms of yield, DPPH, TFC, and TSC (considering only the ethanolic traditional procedures). MHG gave superior results in term of TPC, TAC, and lipase inhibition, while the traditional methods appeared to be better performing in terms of xanthine oxidase and *α*-glucosidase inhibition (Figure 4). These results suggest that the two extraction methodologies provided a different extraction ability towards different compounds and, consequently, their products can present a different suitability according to the market considered.

## 3. Materials and Methods

### 3.1. Plant Material

In June 2020, ripe black mulberry (*M. nigra*) fruits were harvested from local mulberry trees in Camerino, Macerata, Italy. Prof. Filippo Maggi certified the botanical identity of the plant samples, and the voucher specimen was deposited under the codex CAME 28,448 in the Herbarium Camerinensis, which is part of the University of Camerino’s School of Biosciences and Veterinary Medicine, Camerino, Italy. Until extraction, samples were kept in a freezer at −18 °C. The fruits’ average moisture content was 77.21 ± 0.5% and was determined with an electric oven at 110 °C until constant weight was obtained.

### 3.2. Chemicals

#### 3.2.1. HPLC Analysis

The 36 analytical standards were obtained from Sigma-Aldrich (Milan, Italy), except for delphinidin-3,5-diglucoside chloride, petunidin-3-glucoside chloride, cyanidin-3-glucoside chloride, delphinidin-3-galactoside chloride, malvidin-3-galactoside chloride, kaempferol-3-glucoside, and quercetin-3-glucoside, which were supplied by PhytoLab (Vestenbergsgreuth, Germany). Single stock solutions of each component were made by dissolving pure analytes in methanol and storing them at 4 °C until analysis, except for anthocyanins, which were maintained at −18 °C until analysis. Working solutions of the standards at different concentrations were made every day by dilution of the stock solutions with methanol. All solvents used for chromatographic purposes were HPLC grade. The 99% Formic acid was supplied by Merck (Darmstadt, Germany). HPLC-grade methanol was purchased from Sigma-Aldrich (Milano, Italy). Deionized water (>18 MΩ cm resistivity) was further purified using a Milli-Q SP Reagent Water System (Millipore, Bedford, MA, USA). All solutions and solvents were filtered by a 0.2-μm polyamide filter purchased from Sartorius Stedim (Goettingen, Germany). All samples were filtered before HPLC analysis, by using Phenomenex 0.2-μm syringeless filters.

#### 3.2.2. Biological Studies

The 1-diphenyl-2-picrylhydrazyl (DPPH) dye, Folin–Ciocalteu reagent, and gallic acid, were provided by Sigma-Aldrich (Milan, Italy). Lipase, *α*-glucosidase, xanthine, xanthine oxidase, and orlistat were obtained from Sigma-Aldrich (Madrid, Spain). Acarbose was acquired through Cymit Quimica (Barcelona, Spain) All other reagents, unless indicated, were purchased from Sigma (St. Louis, MO, USA).

### 3.3. Extraction Procedures

#### 3.3.1. Microwave Hydrodiffusion and Gravity (MHG) Extraction

**Figure 5:**
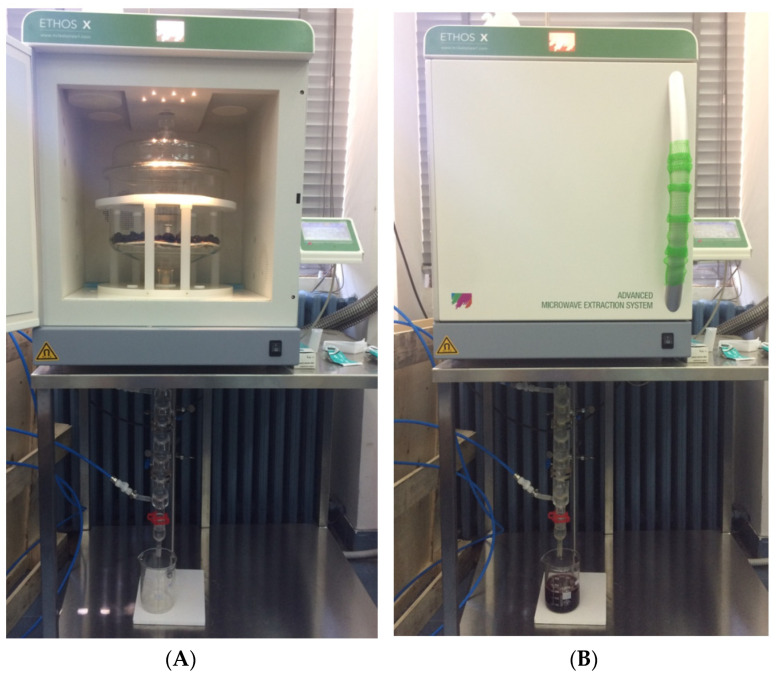
ETHOS X advanced microwave extraction system (MHG) before (**A**) and during extraction (**B**).

A Milestone ETHOS X advanced microwave extraction system (Milestone, Italy) was used for MHG (Figure 5). This is a 2.45-GHz multimode microwave reactor possessing two magnetrons, giving a maximum delivered power of 1800 W (2 × 950 W), and a temperature monitoring infrared sensor. At atmospheric pressure, the experiments were performed in a 5-L reactor made up of Pyrex glass with a glass top (Figure 5). ‘Flavors’ setup’ was used to configure the system. MHG extraction was carried out as follows: 500 g of fresh, intact fruits were placed in a Pyrex reactor and heated at a fixed power density without the addition of any solvent or water. Microwaves interacted directly with biological water, allowing bioactive compounds trapped inside the mulberry fruits cells to be released. The crude extract or juice containing these components thus diffused out of the fruit cells naturally and moved down under the effect of gravity on a spiral condenser that was cooled to 8 °C outside the microwave chamber where it condensed. In a graduated beaker, the crude juice was continually collected. The extraction was carried out according to the conditions proposed by the central composite design (Table 3). The crude juice was collected and freeze-dried after extraction.

#### 3.3.2. Conventional Solvent Extraction

Ten g of fresh fruits (finely cut) were extracted with 50 mL solvent (three replicates). Two different solvents were used: 70% ethanol acidified with formic acid (1.5%) and water (100%). The extraction mixture was kept at 25 °C for 1 h either using a magnetic stirrer (M2-A, Argo Lab, Carpi, MO, Italy) or an ultrasonic bath (FALC ultrasonic bath, Treviglio, Italy). The same fruit sample was extracted until exhaustion (three times). Then, the extract was filtered and concentrated using a rotavapor at 40 °C and then lyophilized until a semisolid paste was obtained. The lyophilized extracts were kept at −18 °C until analysis.

### 3.4. Design of the Experiments (DoE)

#### 3.4.1. Central Composite Design

The investigation of the effect of the experimental MAE factors, microwave power (MP) and extraction time (ET) on the mulberry juice features (yield, total sugar content, polyphenols, flavonoids and anthocyanins content, radical scavenging activity, enzymes’ inhibitions assays), was performed by applying a response surface methodology (RSM) design, the central composite design (CCD). The CCD for two factors was composed by a total of 11 runs, constituted by four factorial experiments (factors’ values were set to the factorial levels +1 and −1), four axial experiments (factors’ values were set to the axial levels +α and +α combined with the central level 0), and three replicates of the central experiment (factors’ values were set to the central level 0). The complete list of the runs required by the CCD is reported in Table 3. The absolute values of the factorial levels was chosen by the authors according to their previous experience with MAE [20], evaluating the preliminary runs requiring the more extreme conditions and, consequently, potential issues such as product burning or absence of product. Table 3 shows all the 11 extractions’ runs of MHG, together with the accompanying coded and uncoded variables. The following characteristics were assigned to each extraction run:

(1) Extraction yield %, calculated as the weight of lyophilized extract per dry weight of fruit.
(2) Total phenolic content (TPC), determined as reported in Section 3.6.
(3) Total flavonoid content (TFC), determined as reported in Section 3.7.
(4) Total anthocyanin content (TAC), determined as reported in Section 3.8.
(5) Total sugar content (TSC), determined as reported in Section 3.9.
(6) α-Glucosidase inhibition, determined as reported in Section 3.10.
(7) Lipase inhibition, determined as reported in Section 3.11.
(8) Xanthine oxidase inhibition, determined as reported in Section 3.12.1.
(9) DPPH radical scavenging, determined as reported in Section 3.12.2.

These parameters represent the responses of the design in the DoE terminology, while the microwave irradiation power and extraction time were the design factors or variables. Multilinear regression using a full quadratic model was used to assess the results of each single response for all of the CCD runs:

(1) $$y=\beta_{0}+\overset{n}{\underset{i=1}{\sum }}\;\beta_{i}\cdot x_{i}+\overset{n}{\underset{i=1}{\sum }}\;\beta_{ii}\cdot x_{i}^{2}+\underset{i<j}{\sum }\;\beta_{ij}\cdot x_{i}x_{j}$$

where *y* is the response, *β*_0_ is the model constant, *β_i_* is the coefficient corresponding to the variables *x_i_* (linear terms), *β_ii_* is the coefficients associated with the variables *x_i_*^2^ (quadratic terms), and *β_ij_* is the coefficients associated with the variables *x_i_x_j_* (first-order interaction terms).

All the full quadratic models that had been determined (one for each response) were submitted to a model reduction procedure using stepwise regression operating in backward elimination mode, to increase the precision of the estimated coefficients of the kept variables, reduce mean square error, and, in general, adhere to the parsimony principle [30]. Evaluation of the Mallows’ Cp statistic, the predicted coefficient of multiple determination (R^2^_pred_), and the adjusted coefficient of multiple determination (R^2^_adj_) were used to select the best model among all those obtained from the stepwise regression [30]. ANOVA, coefficient, and residual analysis were used to evaluate the final models. The Minitab 18 evaluation statistical software was used for model fitting, reduction, selection, and analysis.

#### 3.4.2. Optimization and Validation

The MHG extraction process was optimized using the desirability technique, a multiple responses’ optimization procedure. For all responses, a partial desirability function (Dp) was used to maximize or minimize their values. The geometric mean of all Dp for all combinations of the two examined factors, i.e., MP, and ET, was used to generate the composite desirability function D. D is a number that ranges from 0 to 1; 0 means that at least one response is entirely unsatisfactory, while 1 means that all the responses are completely satisfactory. A surface map of D was created and utilized to visualize the regions with the highest values of D (closest to 1).

The best sets of experimental conditions (run 12) were identified, and the predicted responses were determined, along with their 95% prediction intervals. Run 12 was carried out and analyzed as all the runs of the CCD. The obtained results were then compared with the desirability predictions.

### 3.5. HPLC-ESI-MS/MS Analysis of the Validation MHG Run

Following our previously published method [25], we quantified 36 phenolic compounds in the validated microwave extraction run (MHG 12). Five mg of lyophilized extract were reconstituted in 5 mL of H_2_O (1 mg/mL), and the solution was filtered through a 0.2 µm syringeless filter before being injected into the HPLC-MS/MS apparatus. HPLC-MS/MS experiments were carried out with an Agilent 1290 Infinity series and an Agilent Technology (Santa Clara, CA, USA) Triple Quadrupole 6420 equipped with an electrospray ionization (ESI) source working in positive and negative ionization modes. Using Optimizer Software (Agilent), the MS/MS parameters of each analyte were tuned in flow injection analysis (FIA). Phenomenex’s analytical column, namely, Synergi Polar–RP C18 (250 × 4.6 mm, 4 μm) was used to separate the target chemicals (Chesire, UK), and a Polar RP security guard cartridge (4 × 3 mm ID) preceded the column. In gradient elution mode, the mobile phase was a combination of (A) water and (B) methanol, both containing formic acid 0.1%, at a flow rate of 0.8 mL/min. The mobile phase’s composition changed as follows: 0–1 min, isocratic condition, 20% B; 1–25 min, 20–85% B; 25–26 min, isocratic condition, 85% B; 26–32 min, 85–20% B. Then, 2 μL of extracts were injected. The column temperature was set at 30 °C, and the drying gas temperature in the ionization source was kept at 350 °C. The gas flow was 12,000 mL/min, the nebulizer pressure was 55 psi, and the capillary voltage was 4000 V. Dynamic-multiple reaction monitoring mode was used to detect analytes, and the areas of peaks of the most abundant product ions were integrated for quantification.

### 3.6. Total Phenolic Content (TPC)

The TPC was carried out by the Folin–Ciocalteu method, described by [31,32], with few modifications. Briefly, 0.5 mL of extract solution (1 mg/mL) was introduced into test tubes, then 2.5 mL of Folin–Ciocalteu reagent solution (diluted 10 times in water) and 7 mL of 7.5% Na_2_CO_3_ solution (dissolved in water) were added. The mixture of the reaction was allowed to stand in the dark at room temperature for 2 h and absorption was recorded spectrophotometrically at 765 nm. Quantification of TPC in the extracts was made using gallic acid standard to establish a calibration curve. TPC was expressed as gallic acid equivalents per 100 g of dry extract (mg GAE/100 g). The results were calculated as the average of three experiments.

### 3.7. Total Flavonoid Content (TFC)

The TFC was carried out by the spectrophotometric method, described previously [33], in which 0.5 mL of extract solution (1 mg/mL), 0.15 mL of 0.5 M NaNO_2_, 3.2 mL of 30% methanol (*v*/*v*), and 0.15 mL of 0.3 M AlCl_3_ were mixed. Then, 1 mL of NaOH (1 M) was added after 5 min. The reaction mixture was mixed well and the absorbance using UV spectrophotometer was recorded against a blank reagent at 506 nm. The standard calibration curve for TFC was made using rutin standard solutions under the same aforementioned procedure. The TFC was calculated as mg of rutin equivalents per 100 g dry extract (mg RE/100 g).

### 3.8. Total Anthocyanin Content (TAC)

The TAC was determined by using the pH differential method, reported previously [34]. The extracts were simultaneously diluted (1:10) by using the buffers of 0.025 mol/L potassium chloride at pH = 1 and 0.4 mol/L sodium acetate at pH = 4.5, respectively. Then, the absorbances of the diluted extracts at both pH = 1 and pH = 4.5 were recorded at 510 and 700 nm, respectively. The TAC was estimated using the following formula:

TAC = [(A_510 nm_ − A_700 nm_) pH_1.0_ − (A_510 nm_ − A_700 nm_) pH_4.5_] MW × TV × DF × 1000/(ε × L × SW) (2)

where A is the absorbance, MW is the molecular weight of cyanidin-3-glucoside, which equals to 449.2 g/mol^−1^, TV represents the extract total volume, DF indicates the dilution factor, ε is the extinction coefficient, which equals to 22,400 L/(mol × cm), L is the cuvette length of 1 cm, and SW represents the weight of sample or starting material. The results were calculated as mg of cyanidin-3-glucoside equivalent (CGE) per 100 g dry extract.

### 3.9. Total Sugar Content (TSC)

The total sugar content in extracts of mulberry fruits was estimated by the method described by Fei et al. [35] with some modifications. One mL of extract solution (1 mg/mL) and 0.5 mL of phenol solution (5% in H_2_O) were mixed in a test tube; then, 3.5 mL of H_2_SO_4_ (96%) were added and mixed thoroughly. Then, the test tube was cooled for 20 min, and the absorbance was measured at 490 nm. All samples were done in triplicate. The results were expressed as mg of glucose equivalents for 100 g dry extract using glucose calibration curve.

### 3.10. α-Glucosidase Inhibition

The inhibition of α-glucosidase enzyme by extracts was measured by using a 96-well microplate reader, and the absorbances were measured spectrophotometrically at 405 nm [36,37]. Each well contained 50 µL of extract and 100 µL of enzyme (1 U/mL) dissolved in buffer (12.5 mM Na_2_HPO_4_, 3.3 mM NaH_2_PO_4_; pH = 6.9) and then incubated at room temperature for 10 min. Afterwards, 50 µL of pNPG (3 mM) were added and incubated for 15 min at 37 °C. Finally, the absorbance was recorded. Acarbose was used as positive control under the same aforementioned conditions.

### 3.11. Lipase Inhibition

The ability of the tested black mulberry extracts to inhibit lipase enzyme was evaluated in 96-well plates [38]. Forty µL of extract solution at different concentrations were mixed with 40 µL of enzyme (2.5 mg/mL in 0.1 M phosphate buffer, pH 7.0), previously centrifugated at 2000× *g* for 7 min, and 20 µL of substrate solution (10 mM of p-NPB). Then, incubation at 37 °C for 10 min was done. The absorbance was measured spectrophotometrically at 405 nm. Orlistat was used as positive control under the same conditions.

### 3.12. Free Radical Scavenging Activity

The capacity of the extract to scavenge free radicals was performed by two methods, using DPPH [39] and through the xanthine/xanthine oxidase reaction [40], both using gallic acid as positive control.

#### 3.12.1. Xanthine/Xanthine Oxidase Inhibition

The xanthine/xanthine oxidase reaction, consisting of 90 µM xanthine, 16 mM Na_2_CO_3_, and 22.8 µM NBT, was dissolved in a phosphate buffer 18 mM (pH = 7) to reach a volume of 240 µL. Then, 30 µL of extract and 30 µL of xanthine oxidase (168 U/L) were added to start the reaction. The reaction mixture was incubated for 2 min at 37 °C. Then, the absorbance was measured at 560 nm and the activity of the extracts was determined by the transformation of NBT to the blue chromogen dye by the superoxide radical (O_2_^−^).

#### 3.12.2. DPPH Radical Scavenging

One hundred fifty µL of a DPPH methanolic solution (0.04 mg/mL) were added to 150 µL of the sample at different concentrations dissolved in methanol. The plate was measured at 517 nm after an incubation time of 30 min under dark conditions.

### 3.13. Moisture Content

Moisture content was determined with an electric oven at 110 °C until constant weight was achieved. The average moisture content was 77.21 ± 0.5%. Therefore, the calculation of the extraction yield% of mulberry fruits was based on dry weight (DW).

## 4. Conclusions

The fruit of black mulberry includes a high concentration of phenolic compounds, which may have beneficial health effects. We report, herein, a study of MHG solvent-free extraction of polyphenols and sugars from fresh fruits of black mulberry using a central composite design, with selected responses such as extraction yield, total polyphenol, flavonoid, anthocyanin, and sugar contents, in addition to α-glucosidase inhibition (AGHi), lipase inhibition (Li), xanthine oxidase inhibition (XOi), and DPPH radical scavenging activities as tools to evaluate the best parameters for rapid and efficient extraction of black mulberry. Once we defined the mathematical relationships between the operative parameters and each single response (EY%, TPC, TAC, Li, and XOi), it was possible to optimize the MHG process. The highest desirability values were located in the middle of the experimental domain, specifically in the ranges of 1.5–2.3 W/g and 16–44 min for extraction conditions, namely, the MP and ET, respectively.

The optimum extraction conditions for MP and ET were 1.86 W/g and 31.07 min, respectively, and they were applied in the validation run (run 12); the obtained juice was characterized in terms of the selected responses. The values of EY%, TPC, Li, TAC, and XOi were all within the range of the 95% interval of prediction, suggesting the reliability of the defined models. In addition, the absolute values were always higher than the average ones calculated for the CCD runs, confirming that the validation run performs better than all the other runs if all these features are considered together. The only exception is represented by the TAC value, which is closer to the average value of the CCD runs; the TAC values suddenly declined when the MP reached around 1.8–1.9 W/g. The optimized MHG extract was analyzed for 36 individual polyphenols using HPLC-MS/MS and a total of 23 phenolic compounds were detected. Their contents are consistent with those previously reported in black mulberry and this confirmed the retention of individual polyphenols during the MHG solvent-free extraction. The optimized MHG extract was finally compared with traditional extracts, and performed much better in terms of TPC, TAC, and Li, while the traditional extracts showed better XOi and AGHi. In conclusion, MHG is a valuable tool in the preparation of non-degraded, polyphenol-rich extracts from black mulberry, suggesting its larger use in the pharmaceutical and food industries.

## Figures and Tables

**Table 1:** Best mathematical model for each response and its evaluation parameters: coefficients of determinations (*R*^2^*_adj_* and *R*^2^*_pred_*). Mallows’ Cp statistic and ANOVA results (*p*-values of regression and lack of fit).

Response	Best Model ^a^	*R^2^*	*R^2^_adj_*	*R^2^_pred_*	Mallow’s Cp	*p-*Value regr ^b^	*p-*Value LOF ^b^
EY%	Y = −2.83 + 0.36*P* + 0.519*T* − 1.48*7P*^2^ − 0.006*T*^2^	0.972	0.953	0.872	4.66	***	*
TPC (mg GAE/100 g)	Y = −3393 + 4135P +127.9T − 1343 *P^2^* − 2.61 *T*^2^ *+* 39.4 P*T	0.936	0.873	0.623	6	**	ns
TFC (mg RE/100 g)	Y = 1352 + 99P − 16.4T + 10.76 P*T	0.654	0.506	0.00	2.08	*	ns
TAC (mg CGE/100 g)	Y = 361 + 1008P − 13.01T − 327.6 *P*^2^	0.934	0.902	0.814	5.01	***	*
TSC (mg GE/100 g)	Y = 33,210 − 4034P − 495T + 239 P*T	0.652	0.477	0.00	2.27	ns	**
AGHi IC_50_ (µg/mL)	Y = 442 − 578P + 15.1T +230P2 − 8.9P*T	0.726	0.360	0.00	6.52	ns	**
Li IC_50_ (µg/mL)	Y = 10,447 − 8735*P +* 2260*P*^2^	0.823	0.779	0.625	4.11	**	ns
XOi IC_50_ (µg/mL)	Y = 2045 − 1348*P* − 33.8*T* + 304.7*P*^2^ + 0.455*T*^2^	0.913	0.855	0.58	4.02	**	**
DPPH IC_50_ (µg/mL)	Y = 481.6 − 63.6P − 1.79T	0.598	0.498	0.273	1.37	*	ns

EY%, extraction yield %; TPC, total phenolic content; TFC, total flavonoid content; TAC, total anthocyanin content; TSC, total sugar content; AGHi, α-glucosidase inhibition; Li, lipase inhibition; XOi, xanthine oxidase inhibition; ^a^ The coefficients of the models refer to the the uncoded variables; ^b^ The *p*-value results are indicated as follows: ns, *p* > 0.05; * 0.05 < *p* < 0.01; ** 0.01 < *p* < 0.001; *** *p* < 0.001.

**Table 2:** MHG experimental conditions, desirability, predicted values, and the 95% interval of predictions of the validation run for the tested responses.

Run.	MAE Conditions		Composite Desirability	Responses Optimized with Desirability	Desirability Function	95% Interval of Confidence	95% Interval of Prediction
	Power (W/g)	Time (min)					
12	1.86	31.07	0.83	EY%	Maximize	12.4–14.9	11.1–16.2
				TPC	Maximize	2906–3901	2379–4428
				TAC	Maximize	622–806	525–903
				Li	Minimize	1476–2536	597–3415
				XOi	Minimize	0–98.4	0–240

**Table 3:** The conditions of MHG for all the 11 runs carried out according to the central composite design (CCD). Each single factor’s set is presented as both coded and uncoded variables.

Run.	Point Type ^a^	Coded Variables ^b^		Uncoded Variables		Absolute Values	
		ET (min)	MP (W/g)	ET (min)	MP (W/g)	ET (min)	MP (W)
1	F	−1	−1	15	1	15	500
2	F	−1	1	15	2.4	15	1200
3	F	1	−1	45	1	45	500
4	F	1	1	45	2.4	45	1200
5	A	0	−1.41	30	0.7	30	355
6	A	0	1.41	30	2.7	30	1345
7	A	−1.41	0	8.8	1.7	8.8	850
8	A	1.41	0	51.2	1.7	51.2	850
9	C	0	0	30	1.7	30	850
10	C	0	0	30	1.7	30	850
11	C	0	0	30	1.7	30	850

^a^ The “point type” column defines whether a certain set of experimental conditions represents a factorial (F), axial (A), or central (C) point in the CCD experimental domain. ^b^ The coded variables 1, 1.41, and 0 represent the point type as defined in the “point type” column. The coded variable with value 1.41 represents the α value, that is, the radius of a circle inscribing a square having the length sides equal to 1.

**Table 4:** Contents of phenolic compounds (mg/kg dry extract ± RSD% (*n* = 2)) determined by HPLC-MS/MS in the validated MHG extract.

No.	Compound	Concentration (mg/kg ± RSD%)
Anthocyanins		
1	Delphindin-3,5-diglucoside	nd
2	Delphindin-3-galactoside	nd
3	Cyanidin-3-glucoside	6016.72 ± 3.1
4	Petunidin-3-glucoside	nd
5	Pelargonidin-3-rutinoside	68.52 ± 3.2
6	Pelargonidin-3-glucoside	108.81 ± 4.5
7	Malvidin-3-galactoside	nd
Flavonols		
8	Quercetin	46.49 ± 1.7
9	Rutin	222.9 ± 1.2
10	Isoquercitrin	94.4 ± 2.1
11	Quercitrin	nd
12	Hyperoside	207.67 ± 0.6
13	Isorhamnetin	nd
14	Myricetin	nd
15	Kaempferol	1.35 ± 4.3
16	Kaempferol-3-glucoside	7.85 ± 3.0
Flavan-3-ols		
17	(+)-Catechin	1.92 ± 0.9
18	(-)- Epicatechin	nd
19	Procyanidin A2	nd
20	Procyanidin B2	nd
Dihydrochalcones		
21	Phloridzin	1.41 ± 2.0
22	Phloretin	1.12 ± 1.2
Flavanones		
23	Hesperidin	nd
24	Naringin	nd
Phenolic acids		
25	Neochlorogenic acid	57.5 ± 1.2
26	Chlorogenic acid	565.6 ± 1.7
27	Gallic acid	4.0 ± 0.8
28	*p*-Hydroxybenzoic acid	0.4 ± 1.1
29	3-Hydroxybenzoic acid	nd
30	Caffeic acid	3.9 ± 2.7
31	Vanillic acid	8.0 ± 1.3
32	Syringic acid	2.2 ± 1.12
33	*p*-Coumaric acid	8.8 ± 2.6
34	Ferulic acid	1.6 ± 3.3
35	3,5-Dicaffeoylquinic acid	5.0 ± 2.6
36	Ellagic acid	51.66 ± 1.04

nd, not detected.

## Data Availability

Data are contained within the article and Supplementary Materials.

## Supplementary Materials

The following are available online at https://www.mdpi.com/article/10.3390/ph15010099/s1. Figure S1: Results of the responses not used for the optimization of the validation run. Table S1: HPLC–MS/MS acquisition parameters (dynamic-MRM mode) used for the analysis of the 36 marker compounds. Table S2: Method validation data of the analyzed 36 phenolic compounds by HPLC–MS/MS. Figure S2: HPLC-MS/MS chromatogram of a standard mixture of 36 phenolic compounds plotted as overlapped multiple reaction monitoring (MRM) transition of each analyte.
