# Supplementary material for: Optimization of Solvent-Free Microwave-Assisted Hydrodiffusion and Gravity Extraction of Morus nigra L. Fruits Maximizing Polyphenols, Sugar Content, and Biological Activities Using Central Composite Design
Source: Pharmaceuticals (Basel). 2022 Jan 14;15(1):99. doi: 10.3390/ph15010099 (PMC8780424; doi:10.3390/ph15010099)
Supplement: Supplementary file 1 [file pharmaceuticals-15-00099-s001.zip › pharmaceuticals-1548653-supplementary.pdf]

Supplementary Materials

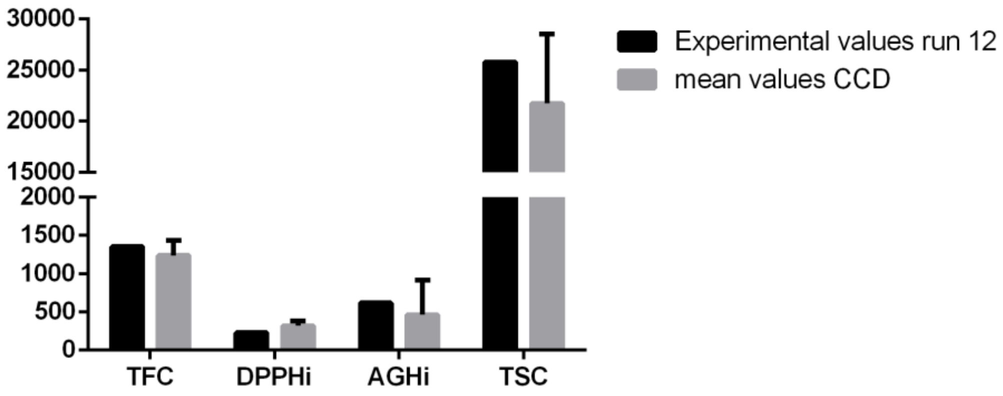

Figure S1. Results of the responses not used for the optimization of the validation run

Table S1. HPLC–MS/MS acquisition parameters (dynamic-MRM mode) used for the analysis of the 36 marker compounds.

No.	Compounds	Precursor ion, *m/z*	Product ion, *m/z*	Fragm-entor, V	Collision energy, V	Polarity	Retention time (Rt, min)	Delta retention time (ΔRt)
1	Gallic acid	169	125.2*	97	12	Negative	6.96	2
2	Neochlorogenic acid	353	191.2*, 179	82	12, 12	Negative	9.52	2
3	Delphinidin-3-galactoside	465.01	303*	121	20	Positive	11.36	2
4	(+)-Catechin	289	245.2*,109.2	131	8, 20	Negative	11.44	2
5	Procyanidin B2	576.99	576.99*, 321.2	160	0, 32	Negative	12.41	2
6	Chlorogenic acid	353	191.2*, 127.5	82	12, 20	Negative	12.42	2
7	*p*-Hydroxybenzoic acid	137	93.2*	92	16	Negative	12.86	2
8	(-)-Epicatechin	289	245.1*, 109.1	126	8, 20	Negative	13.03	2
9	Cyanidin-3-glucoside	449	287.3*, 255.6	121	20, 20	Positive	13.14	2
10	Petunidin-3-glucoside	479.01	317*, 302	121	20, 44	Positive	13.26	2
11	3-Hydroxybenzoic acid	137	93.2*	88	8	Negative	13.59	2
12	Caffeic acid	179	135.2*, 134.1	92	12, 24	Negative	13.65	2
13	Vanillic acid	167	152.4*, 108.1	88	12, 20	Negative	14.32	2
14	Pelargonidin-3-glucoside	433.01	271*, 121	116	24, 50	Positive	14.52	2
15	Pelagonidin-3-rutinoside	579.01	271*	145	32	Positive	14.56	2
16	Malvidin-3-galactoside	493.01	331*, 315.1	121	20, 50	Positive	14.64	2
17	Syringic acid	196.9	182.2*, 121.2	93	8, 12	Negative	15.28	2
18	Procyanidin A2	575	575*, 285	170	0, 20	Negative	16.18	2
19	*p*-Coumaric acid	163	119.2*, 93.2	83	12, 36	Negative	16.70	2
20	Ferulic acid	193	134.2*, 131.6	83	12, 8	Negative	17.10	2
21	3,5-Dicaffeoylquinic acid	514.9	353.1*, 191	117	8, 28	Negative	17.61	2
22	Rutin	609	300.2*, 271.2	170	32, 50	Negative	17.73	2
23	Hyperoside	465.01	303*, 61.1	97	8, 50	Positive	18.33	2
24	Isoquercitrin	463	271.2*, 300.2	155	44, 24	Negative	18.36	2
25	Delphinidin-3,5-diglucoside	462.9	300.1*	165	24	Negative	18.38	2
26	Phloridzin	435.39	273*, 167	155	8, 28	Negative	18.83	2
27	Quercitrin	446.99	300.2*, 301.2	160	24, 16	Negative	19.61	2
28	Myricetin	316.99	179.1*, 182	150	16, 24	Negative	19.61	2
29	Naringin	578.99	271.3*, 151.3	170	32, 44	Negative	19.62	2
30	Kaempferol-3-glucoside	447	284.2*, 255.2	170	24, 40	Negative	19.77	2
31	Hesperidin	611.01	303*, 334.8	112	20, 12	Positive	20.19	2
32	Ellagic acid	301	301*, 229	170	0, 24	Negative	21.41	2
33	Quercetin	300.99	151.2*, 179.2	145	16, 12	Negative	21.87	2
34	Phloretin	272.99	167*, 123	116	8, 20	Negative	22.30	2
35	Kaempferol	287.01	153*, 69.1	60	36, 50	Positive	23.84	2
36	Isorhamnetin	314.99	300.2*, 196.1	145	16, 4	Negative	24.57	2

* These product ions were used for quantification.

Table S2. Method validation data of the analysed 36 phenolic compounds by HPLC–MS/MS

No.	Compounds	Conc. range (mg/L)	$R^{2\,a}$	LOD (mg/L)[b]	LOQ (mg/L)[c]	Repeatability (RDS[d] %, *n=3*)	
						Intraday[e]	Interday[f]
1	Gallic acid	0.005-10	0.9956	0.003	0.009	2.62	3.81
2	Neochlorogenic acid	0.005-10	0.9984	0.002	0.006	0.22	2.36
3	Delphinidin-3-galactoside	0.005-10	0.9988	0.0016	0.005	0.69	3.35
4	(+)-Catechin	0.005-10	0.9977	0.0026	0.008	0.92	4.64
5	Procyanidin B2	0.005-10	0.9961	0.0033	0.01	0.74	0,40
6	Chlorogenic acid	0.005-10	0.9993	0.0033	0.01	0.65	0,77
7	*p*-Hydroxybenzoic acid	0.005-10	0.9980	0.0027	0.0083	0.94	2.23
8	(-)-Epicatechin	0.005-10	0.9977	0.0025	0.0075	0.93	1.34
9	Cyanidin-3-glucoside	0.005-10	0.9967	0.0023	0.0070	0.49	3.41
10	Petunidin-3-glucoside	0.005-10	0.9970	0.001	0.0029	0.63	2.25
11	3-Hydroxy benzoic acid	0.005-10	0.9992	0.0024	0.0072	0.80	4.13
12	Caffeic acid	0.005-10	0.9985	0.0015	0.0045	0.13	1.89
13	Vanillic acid	0.005-10	0.9943	0.0033	0.01	0.71	4.73
14	Pelargonidin-3-glucoside	0.005-10	0.9979	0.0009	0.0027	0.79	3.79
15	Pelagonidin-3-rutinoside	0.005-10	0.9976	0.0012	0.0036	1.00	4.54
16	Malvidin-3-galactoside	0.005-10	0.9954	0.001	0.0030	0.71	3.22
17	Syringic acid	0.005-10	0.9997	0.0032	0.01	0.66	4.43
18	Procyanidin A2	0.005-10	0.9998	0.0033	0.0099	0.58	1.26
19	*P*-Coumaric acid	0.005-10	0.9990	0.0031	0.0093	0.87	0.96
20	Ferulic acid	0.005-10	0.9959	0.0013	0.0039	0.82	2.93
21	3,5-Dicaffeoylquinic acid	0.005-10	0.9999	0.0037	0.0111	0.82	1.83
22	Rutin	0.005-10	0.9985	0.003	0.009	0.89	1.30
23	Hyperoside	0.005-10	0.9972	0.002	0.006	0.90	4.09
24	Isoquercitrin	0.005-10	0.9999	0.0025	0.008	0.84	1.78
25	Delphinidin-3,5-diglucoside	0.005-10	0.9998	0.0019	0.006	0.69	2.89
26	Phloridzin	0.005-10	0.9995	0.0022	0.0066	0.27	1.01
27	Quercitrin	0.005-10	0.9990	0.003	0.009	0.91	4.00
28	Myricetin	0.005-10	0.9992	0.0026	0.008	0.35	1.03
29	Naringin	0.005-10	0.9970	0.0017	0.0051	0.26	3.93
30	Kaempferol-3-glucoside	0.005-10	0.9950	0.0016	0.005	0.73	1.40
31	Hesperidin	0.005-10	0.9994	0.0017	0.0051	0.12	2.28
32	Ellagic acid	0.005-10	0.9999	0.0033	0.01	0.86	0.92
33	Quercetin	0.005-10	0.9998	0.0015	0.0045	0.95	1.35
34	Phloretin	0.005-10	0.9951	0.0006	0.002	0.79	1.26
35	Kaempferol	0.005-10	0.9989	0.0026	0.008	0.86	1.03
36	Isorhamnetin	0.005-10	0.9987	0.0004	0.0012	0.77	1.80

[a] R^2: Coefficient of determination
[b] LODs: (limit of detection) = ratio of signal to noise (S/N) = 3
[c] LOQs: (limit of quantification) = ratio of signal to noise (S/N) = 10
[d] RSD: Relative standard deviation.
[e] Intraday: Obtained by analysing 3 times a mixture standard solution at a concentration of 1 mg/L on the same day.
[f] Interday: Obtained by analysing standard solutions at a concentration of 1 mg/L during 3 consecutive days.

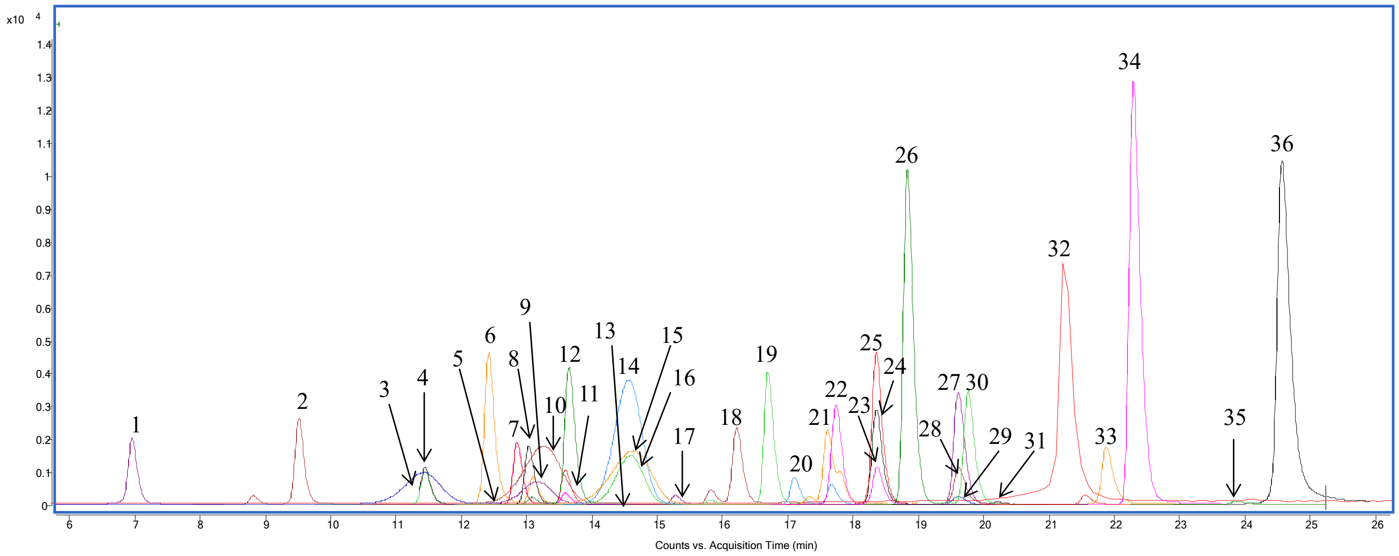

Figure S2. HPLC-MS/MS chromatogram of a standard mixture of 36 phenolic compounds plotted as overlapped multiple reaction monitoring (MRM) transition of each analyte. (1) Gallic acid, (2) Neochlorogenic acid, (3) Delphinidin-3-galactoside, (4) (+)-Catechin, (5) Procyanidin B2, (6) Chlorogenic acid, (7) *p*-Hydroxybenzoic acid, (8) (-)-Epicatechin, (9) Cyanidin-3-glucoside, (10) Petunidin-3-glucoside, (11) 3-Hydroxybenzoic acid, (12) Caffeic acid, (13) Vanillic acid, (14) Pelargonidin-3-glucoside, (15) Pelagonidin-3-rutinoside, (16) Malvidin-3-galactoside, (17) Syringic acid, (18) Procyanidin A2, (19) *p*-Coumaric acid, (20) Ferulic acid, (21) 3,5-Dicaffeoylquinic acid, (22) Rutin, (23) Hyperoside, (24) Isoquercitrin, (25) Delphinidin-3,5-diglucoside, (26) Phloridzin, (27) Quercitrin, (28) Myricetin, (29) Naringin, (30) Kaempferol-3-glucoside, (31) Hesperidin, (32) Ellagic acid, (33) Quercetin, (34) Phloretin, (35) Kaempferol, (36) Isorhamnetin.
